# Optimal surgical timing after high-altitude de-adaptation: day-30 post-descent marks physiologic recalibration and improved small bowel repair in rats

**DOI:** 10.3389/fphys.2026.1742306

**Published:** 2026-02-09

**Authors:** Yizhi Yue, Xiaohua Wang, Yaning Song, Yi Sun, Lin Xue, Guangyu Chen, Ze Feng, Guode Luo, Tao Wang

**Affiliations:** 1 School of Life Science and Engineering, Southwest Jiaotong University, Chengdu, Sichuan, China; 2 Department of General surgery, The General Hospital of Western Theater Command, Chengdu, Sichuan, China

**Keywords:** high-altitude de-adaptation, inflammatory response, intestinal repair, oxidative stress, surgical timing

## Abstract

**Background:**

High-altitude de-adaptation following rapid transition from chronic hypoxia to normoxia has been associated with increased postoperative risk, yet its temporal physiological features and impact on intestinal repair remain poorly defined.

**Methods:**

Male Sprague–Dawley rats (n = 84) were exposed to simulated high altitude (5,000 m) for 90 days and then relocated to normoxia. Standardized small bowel rupture repair was performed at 1, 10, 20, 30, 40, 50, or 60 days after relocation. Hypoxia adaptation and reversibility were assessed using arterial oxygen saturation, hematological indices, hypoxia-responsive molecular markers, respiratory rate, body weight, and behavior. Postoperative outcomes were evaluated 10 days after surgery, including inflammatory cytokines, oxidative stress markers, immune cell infiltration, and histopathology.

**Results:**

Chronic hypoxia induced a stable hypoxia-adapted state characterized by reduced oxygen saturation, enhanced erythropoiesis, increased respiratory rate, and upregulation of intestinal HIF-1α and vascular endothelial growth factor, all of which progressively normalized after return to normoxia and resolved by approximately 30 days. Perioperative survival did not differ among groups. In contrast, systemic inflammatory cytokines and lipid peroxidation peaked at day 1 post-relocation and declined to nadir levels by day 30. This period was marked by reduced macrophage infiltration, peak fibroblast density, and more organized granulation tissue and collagen deposition.

**Conclusion:**

The duration of high-altitude de-adaptation is closely associated with intestinal repair quality. Approximately 30 days of normoxic re-acclimation correspond to coordinated resolution of hypoxia-related physiological perturbations and optimized tissue repair, identifying a critical post-relocation window relevant to surgical timing after descent from high altitude.

## Introduction

High-altitude adaptation is a multifaceted physiological process characterized by coordinated systemic responses to chronic hypoxia. Extensive studies have elucidated molecular and cellular mechanisms underlying this adaptation, including enhanced erythropoiesis, angiogenic remodeling, and metabolic reprogramming in response to sustained reductions in oxygen availability (Murray et al., 2018; Ferraretti et al., 2024; Mallet et al., 2023).

In contrast, the reverse process, commonly referred to as high-altitude de adaptation, remains comparatively underexplored despite its growing clinical relevance in the context of increasing population mobility. High-altitude de-adaptation is defined as a maladaptive pathophysiological response induced by rapid transition from chronically hypoxic environments to normoxic low-altitude conditions. This process is characterized by dysregulated inflammatory signaling, exacerbated oxidative stress, and consequent impairments in multi-organ function (Zhou et al., 2012; Richalet, 2021; Jiang et al., 2022). Clinical observations conducted at tertiary care centers located in low-altitude regions consistently indicate a heightened incidence of postoperative morbidity among long-term high-altitude residents who undergo abdominal surgery shortly after relocating to these lower altitudes (Zhu et al., 2023; Shang et al., 2022). These complications include delayed wound healing, anastomotic leakage, and heightened systemic inflammatory responses (Plancher et al., 2025; Chen et al., 2022). Collectively, these findings suggest that physiological instability associated with de adaptation may interact with surgical trauma, thereby compromising postoperative recovery.

At present, perioperative management guidelines lack evidence based recommendations tailored to this population, highlighting a critical gap in defining optimal surgical timing following high-altitude relocation. Although hypoxia reoxygenation injury models have provided mechanistic insights into oxidative stress and inflammatory cascades, their translational relevance remains limited by the absence of systematic integration between de adaptation timelines and surgical outcomes. Mechanistically, abrupt reoxygenation following chronic hypoxia exacerbates mitochondrial reactive oxygen species (ROS) production, depleting antioxidant reserves such as superoxide dismutase (SOD) while elevating lipid peroxidation markers like malondialdehyde (MDA), thereby destabilizing redox equilibrium (Bai et al., 2022; Shuang et al., 2023; Li et al., 2016). Concurrently, surgical trauma exacerbates the production of pro-inflammatory cytokines, such as TNF-α and IL-17, as well as acute-phase proteins like CRP. This upregulation impairs the macrophage-mediated clearance of cellular debris and fibroblast-driven tissue remodeling, both of which are crucial processes for intestinal repair (Ueno et al., 2023; Dobson et al., 2021; Chalkidi et al., 2022; Han et al., 2021). In addition, preclinical studies indicate that hypoxia reoxygenation impairs endothelial barrier function and delays epithelial restitution in gastrointestinal tissues, thereby synergizing with surgical stress to prolong mucosal injury (Bai et al., 2022; Wang et al., 2021; Feng et al., 2020).

Despite these advances, no prior investigations have systematically mapped de-adaptation duration to surgical stress resilience or identified recovery thresholds for mitigating postoperative sequelae. To address this, we established a controlled rat model of high-altitude de-adaptation, simulating human relocation through prolonged hypobaric hypoxia exposure followed by normoxic reacclimatization. Small bowel rupture repair surgeries were performed at strategic intervals post-relocation to evaluate temporal trends in inflammatory biomarkers, oxidative stress parameters, and histopathological repair indices. This study aims to establish an evidence-based timeframe for minimizing postoperative complications in high-altitude populations by correlating the duration of de-adaptation with surgical recovery metrics.

## Materials and methods

**FIGURE 1:**
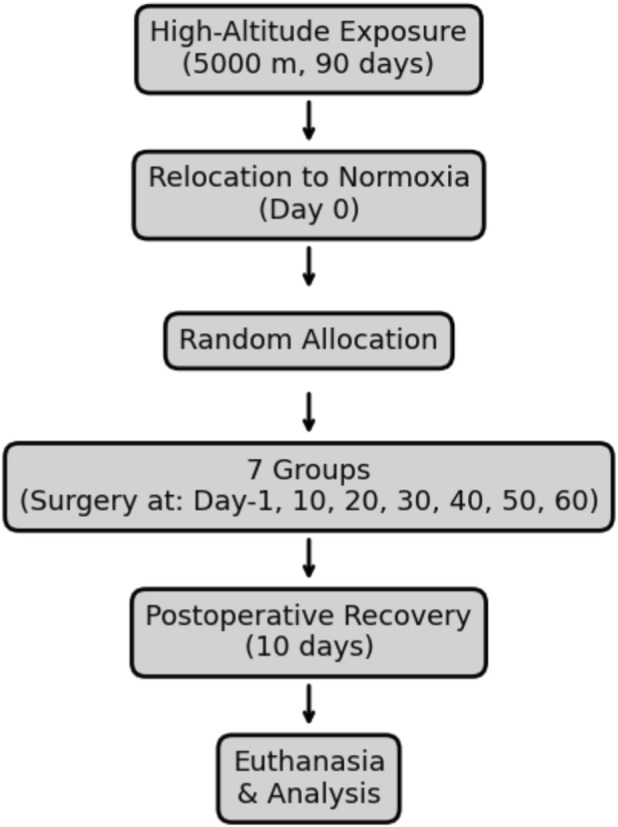
Experimental flowchart of study design.

All animal procedures were conducted in accordance with the ethical standards and regulations of the General Hospital of Western Theater Command. The study protocol received approval from the Institutional Animal Care and Use Committee (IACUC) of the General Hospital of Western Theater Command (Approval No. 2024EC2-ky011). The flowchart of the study is shown in Figure 1.

### Animals and housing

A total of 84 adult male Sprague-Dawley (SD) rats (weight: 300–350 g; supplier: Hunan Slack Jingda Experimental Animal Co., Ltd., Changsha, China; License No. SCXK [Hunan] 2019-0004) were used. Animals were housed in groups of 4–5 per ventilated polycarbonate cage (Allentown LLC, United States), provided with aspen chip bedding and nesting material, and maintained in an environmentally controlled facility (temperature: 21.4 °C ± 0.1 °C; humidity: 49.5% ± 0.05%) on a reversed 12-h light/dark cycle. Rats had *ad libitum* access to irradiated chow (LabDiet 2918, PMI Nutrition International, United States) and reverse-osmosis purified water throughout the study.

### High-altitude de-adaptation model

Following a 7-day acclimatization period in the general animal facility of the Experimental Center of the General Hospital of the Western Theater Command of the Chinese People’s Liberation Army, rats were transferred to a computer-controlled hypobaric hypoxia chamber (HPPC-01, China). The chamber simulated a high-altitude environment of 5,000 m (FiO_2_ 10.8%, barometric pressure 404 mmHg) for 90 consecutive days.

After completion of hypoxic exposure, animals were relocated to normoxic conditions at low altitude (50 m; FiO_2_ 20.9%). Rats were then randomly assigned, using a Latin-square design, to seven surgical cohorts (n = 12 per group) according to the timing of post-relocation intervention: day 1 (24 h post-relocation), day 10, day 20, day 30, day 40, day 50, and day 60.

The selected post-relocation timepoints were designed to capture distinct physiological stages of high-altitude de-adaptation. These included the acute reoxygenation phase (day 1), intermediate transitional phases characterized by residual hypoxia-related adaptations (days 10 and 20), and a late re-acclimation phase in which systemic and molecular hypoxia signatures typically return to baseline levels (day 30). Additional later timepoints (days 40–60) were incorporated to determine whether prolonged normoxic re-acclimation confers further physiological or biological benefits beyond initial stabilization.

### Physiological validation of chronic high-altitude exposure

To confirm that prolonged hypobaric hypoxia induced stable hypoxia-related physiological adaptations prior to relocation, a subset of rats (n = 6) was randomly selected at three time points: baseline (sea level before chamber exposure), end of high-altitude exposure (day 90 at 5,000 m), and after re-acclimation to normoxia (day 30 post-relocation).

Peripheral arterial oxygen saturation (SpO_2_) was measured noninvasively using a rat-adapted pulse oximetry system (MouseOx Plus, Starr Life Sciences) under light isoflurane anesthesia. Hematological parameters, including hematocrit (Hct) and hemoglobin concentration (Hb), were assessed using an automated hematology analyzer (Mindray BC-5000Vet).

To evaluate molecular hypoxia signaling, jejunal mucosal samples were harvested for Western blot analysis of hypoxia-inducible factor-1α (HIF-1α) and vascular endothelial growth factor (VEGF). Protein expression levels were normalized to β-actin and quantified by densitometry.

Body weight was recorded weekly throughout hypoxic exposure and daily during the first 14 days following relocation. Behavioral activity was semi-quantitatively assessed using open-field locomotion scoring, and resting respiratory rate was measured at baseline, at the end of hypoxia, and during re-acclimation.

Recovery from hypoxia-induced physiological adaptation after descent was defined *a priori* using a composite, surrogate-based criterion. Specifically, animals were considered physiologically re-acclimated when the following conditions were met: (i) SpO_2_ returned to within 95% of baseline sea-level values; (ii) hematocrit and hemoglobin concentrations declined to within ±10% of baseline levels; (iii) hypoxia-responsive molecular markers (HIF-1α and VEGF) were no longer significantly elevated compared with baseline; and (iv) resting respiratory rate, body weight trajectory, and spontaneous locomotor activity returned to baseline ranges.

### Surgical protocol

1. Anesthesia and Perioperative Management: Anesthesia was induced in a plexiglass chamber (30 × 20 × 20 cm) using 2% isoflurane (Baxter Healthcare, Cat. No. 1001936040) vaporized in 100% oxygen at a flow rate of 1 L/min. Loss of righting reflex was confirmed within 3–5 min, after which anesthesia was maintained with 1.5%–2% isoflurane via nosecone (O_2_ flow: 0.8 L/min). Core temperature was maintained at 35 °C–40 °C using Sunbeam heating pads. Meloxicam (Boehringer Ingelheim, Cat. No. not specified) was administered subcutaneously at 1 mg/kg every 24 h for five dayspostoperatively for analgesia.Animals were monitored at least twice daily for signs of pain, distress, decreased grooming, piloerection, or impaired mobility. No rescue analgesia criteria were triggered during the study period.
2. Small Bowel Injury and Repair Model: Under aseptic conditions, a 3-cm midline laparotomy was performed. The jejunum was exteriorized 10 cm distal to the ligament of Treitz. A full-thickness, 50% circumferential excision was created using microsurgical scissors (Fine Science Tools, Cat. No. 15000-08). The intestinal defect was repaired with continuous single-layer closure using 6-0 polyglactin 910 sutures (Ethicon VICRYL, Cat. No. VCP311H). The abdominal wall was closed in two layers (fascia and skin) with interrupted 4-0 polypropylene sutures (Ethicon PROLENE, Cat. No. 8698H).
3. Postoperative Care: After surgery, rats recovered individually in temperature-regulated cages with supplemental oxygen (2 L/min for60 min). Animals were monitored daily for signs of distress or infection.

### Sample collection

On postoperative day 10, all surviving rats were euthanized by intraperitoneal injection of 10% chloral hydrate (Sinopharm Chemical, Cat. No. 10031760; dose: 0.3 mL/100 g body weight) followed by cervical dislocation. Blood samples were collected via abdominal aorta puncture, allowed to clot at room temperature, and centrifuged at 3,000 × g for 15 min at 4 °C. Serum was aliquoted and stored at −80 °C until analysis. Jejunal tissue at the repair site was harvested, with portions fixed in 4% paraformaldehyde (Sinopharm Chemical, Cat. No. 10049618) for histopathology and other portions flash-frozen in liquid nitrogen for molecular assays.

### Biomarker assays

Serum concentrations of tumor necrosis factor-α (TNF-α, Cat. No. FKE50201), interleukin-17 (IL-17, Cat. No. FKE50217), C-reactive protein (CRP, Cat. No. FKE50289), malondialdehyde (MDA, Cat. No. FKE50234), and superoxide dismutase (SOD, Cat. No. FKE50276) were measured using commercial ELISA kits (Fankew, Shanghai, China) according to the manufacturer’s instructions. All samples and standards were assayed in duplicate. Absorbance was read using a microplate reader (BioTek Synergy HTX, United States).

### Immunohistochemistry and cell quantification

Fixed jejunal tissues were embedded in paraffin, and 4-μm sections were prepared. Immunohistochemistry was performed using the Bond-III automated stainer (Leica Biosystems, Germany). Primary antibodies included anti-CD68 (1:200, Abcam, Cat. No. ab125212), anti-vimentin (1:150, Cell Signaling Technology, Cat. No. 5741S), and anti-myeloperoxidase (MPO; 1:100, Abcam, Cat. No. ab9535). Antibody binding was visualized with a DAB chromogen (Leica Biosystems). Cell counts were determined in 30 randomly selected high-power fields (×400 magnification) per specimen using an Olympus BX53 microscope equipped with cellSens Dimension 2.3 software. Two independent, blinded pathologists performed the assessments.

### Histopathological analysis

Paraffin-embedded tissue sections were stained with hematoxylin and eosin (H&E) following standard protocols. Tissue repair quality was evaluated qualitatively and quantitatively by blinded observers.

### Blinding and bias control

To minimize potential bias, investigators involved in physiological measurements, biochemical assays, histological quantification, and data analysis were blinded to group allocation throughout the study. Animals were assigned anonymized identification codes immediately after randomization, which were maintained until completion of all outcome assessments and statistical analyses.

Surgical procedures were performed according to a standardized protocol to reduce procedural variability; surgeons were not involved in postoperative data collection or outcome evaluation. Histological analyses were independently performed by two experienced pathologists who were blinded to experimental groups.

### Statistical analysis

Data normality was assessed using the Kolmogorov–Smirnov test (threshold P > 0.10). Parametric variables are presented as mean ± standard deviation (SD) and compared across groups using one-way analysis of variance (ANOVA). Intergroup multiple comparisons were performed using Tukey HSD *post hoc* testing to control Type I error. Survival differences were assessed using the log-rank test. Statistical significance was defined as two-tailed P < 0.05. All statistical analyses were performed using SPSS version 26.0 (IBM Corp., United States of America). No data were excluded from analysis.

## Results

### Establishment and reversibility of chronic hypoxia–induced physiological adaptations

**FIGURE 2:**
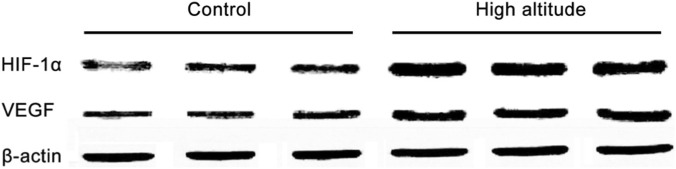
Hypoxia-induced upregulation of intestinal HIF-1α and VEGF expression. Representative Western blottings showing intestinal mucosal expression of HIF-1α and VEGF under normoxic and hypoxic conditions.

Chronic exposure to simulated high altitude (5,000 m for 90 days) induced robust and sustained systemic adaptations consistent with chronic hypoxia. Compared with baseline sea-level conditions, rats at the end of hypoxic exposure exhibited a marked reduction in arterial oxygen saturation (SpO_2_: 97.2% ± 1.1% vs. 82.6% ± 2.4%, P < 0.001), accompanied by pronounced erythropoietic responses, as evidenced by significantly elevated hematocrit (Hct: 44.8% ± 2.3% vs. 59.7% ± 3.1%, P < 0.001) and hemoglobin concentration (Hb: 146 ± 9 g/L vs. 184 ± 11 g/L, P < 0.001). At the molecular level, intestinal mucosal expression of HIF-1α was significantly upregulated under hypoxic conditions, with a parallel increase in VEGF expression, confirming activation of canonical hypoxia-responsive signaling pathways (Figure 2).

**FIGURE 3:**
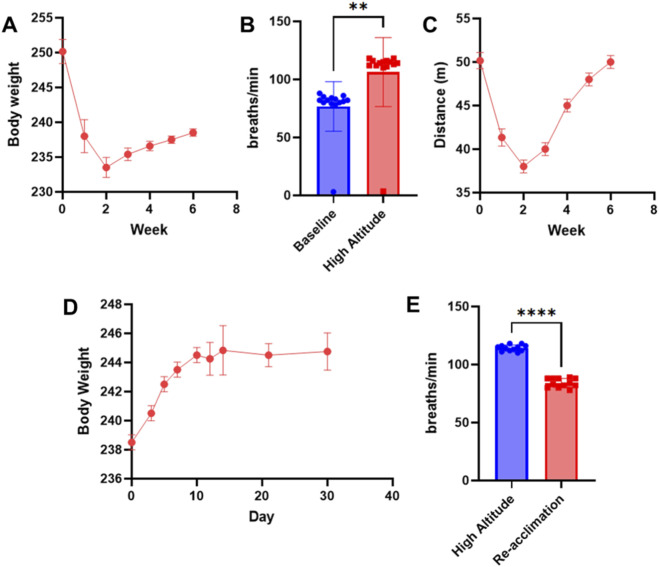
Systemic and behavioral adaptations of rats during hypoxic exposure and subsequent re-acclimation. **(A)** Body weight of rats (n = 12) measured weekly during hypoxic exposure. A transient decrease was observed during the first 2 weeks, followed by stabilization. **(B)** Resting respiratory rate measured at baseline (sea level) and after chronic hypoxic exposure. **(C)** Spontaneous locomotor activity assessed by total distance traveled in the open-field test during hypoxic exposure. **(D)** Body weight changes during normoxic re-acclimation following hypoxic exposure, showing a transient increase during days 7–14 and subsequent stabilization. **(E)** Resting respiratory rate during normoxic re-acclimation, returning to baseline levels within 2 weeks after relocation.

In parallel with these biochemical and molecular alterations, rats displayed characteristic systemic and behavioral adaptations during hypoxic exposure. Body weight showed a transient reduction during the first 2 weeks (maximum decrease 6.4% ± 1.2%), followed by stabilization despite continued hypoxia (Figure 3A), suggesting successful physiological accommodation rather than progressive wasting. Resting respiratory rate increased significantly at high altitude compared with baseline values (82 ± 7 vs. 114 ± 10 breaths/min, P < 0.001), reflecting compensatory cardiorespiratory adaptation (Figure 3B). Open-field testing revealed mildly reduced spontaneous locomotor activity during early hypoxic exposure, which gradually normalized by week 6, indicating behavioral acclimatization rather than persistent distress or functional impairment (Figure 3C).

Following relocation to normoxic conditions, hypoxia-induced physiological alterations exhibited gradual but coordinated reversibility. Arterial oxygen saturation normalized rapidly within 72 h (96.1% ± 1.3%), whereas hematological parameters declined more slowly, returning toward baseline levels by day 30 post-relocation (Hct: 46.9% ± 2.6%; Hb: 152 ± 8 g/L; both P > 0.05 vs. baseline). Consistently, intestinal HIF-1α and VEGF expression decreased progressively during re-acclimation and were indistinguishable from baseline levels by day 30. Body weight demonstrated a transient overshoot during early re-acclimation (days 7–14), followed by stabilization (Figure 3D), while respiratory rate fully normalized within 2 weeks after return to normoxia (Figure 3E).

Collectively, these findings confirm that rats entered the de-adaptation phase from a well-defined, hypoxia-adapted physiological state and that approximately 30 days of normoxic re-acclimation were required for systemic and intestinal hypoxia-associated signatures to fully resolve.

### Effects of de-adaptation duration on postoperative survival

**FIGURE 4:**
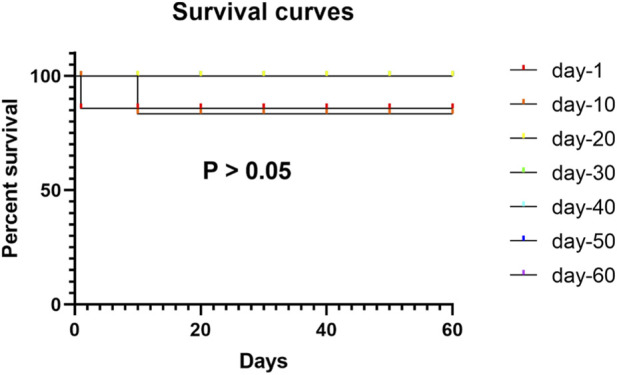
Postoperative survival analysis stratified by duration of low-altitude adaptation.

A total of 84 male Sprague-Dawley rats acclimatized to a simulated high altitude of 5,000 m for 90 days were randomized into seven surgical cohorts according to the duration of normoxic adaptation prior to small bowel repair: day-1, day-10, day-20, day-30, day-40, day-50, and day-60 post-relocation (n = 12 per group). Perioperative survival rates were 91.7% for the day-1 and day-10 groups (each with 1 death) and 100% for all subsequent groups (day-20 through day-60). No statistically significant differences in overall survival were observed among the groups (P > 0.05, log-rank test; Table 1; Figure 4).

**TABLE 1:** Survival and mortality by group.

Group	n (initial)	Deaths	Survival rate (%)
Day-1	12	1	91.7
Day-10	12	1	91.7
Day-20	12	0	100
Day-30	12	0	100
Day-40	12	0	100
Day-50	12	0	100

### Temporal dynamics of systemic inflammatory biomarkers

**FIGURE 5:**
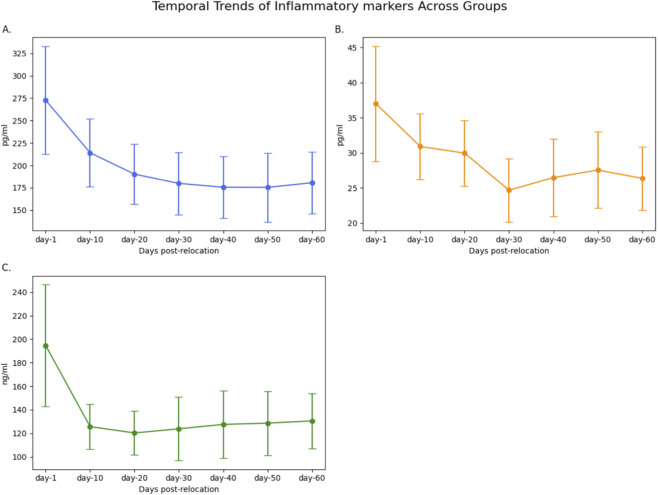
Temporal Trends of Inflamatory Markers Across Groups Temporal trends of TNF-α **(A)**, IL-17 **(B)**, and CRP **(C)** serum levels across groups following relocation from high-altitude to normoxic conditions. Each panel displays mean values ±SD for each biomarker in rats undergoing small bowel repair at different time points post-relocation (day-1, day-10, day-20, day-30, day-40, day-50, and day-60). Inflammatory marker levels declined significantly over time, with nadirs observed at or after day-30, indicating a resolution of the acute inflammatory response associated with high-altitude de-adaptation and surgical intervention.

**FIGURE 6:**
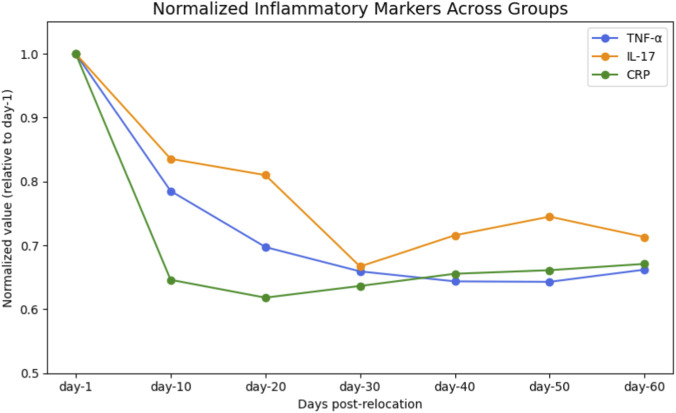
Overlay of Normalized Inflammatory Markers (TNF-α, IL-17, CRP) Across Groups Overlay of serum TNF-α, IL-17, and CRP levels, each normalized to their respective day-1 value, across all experimental groups. Values represent the relative change in inflammatory marker concentration following relocation from high-altitude to normoxia and surgical intervention at each time point (day-1, day-10, day-20, day-30, day-40, day-50, and day-60). All markers showed a progressive decline, reaching their lowest values around day-30 post-relocation, indicative of resolution of systemic inflammation.

**FIGURE 7:**
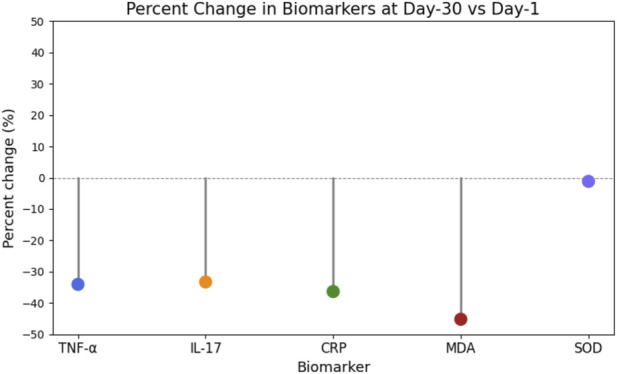
Percent Change in Biomarkers at Day-30 vs. Day-1. Lollipop plot showing the percent change in serum biomarker levels (TNF-α, IL-17, CRP, MDA, and SOD) at day-30 compared to day-1 post-relocation. Negative values indicate a decrease from baseline. Substantial reductions in inflammatory and oxidative stress markers were observed by day-30, while SOD activity remained relatively stable.

Analysis of serum inflammatory markers revealed significant temporal variations across groups. TNF-α, IL-17, and CRP levels exhibited biphasic patterns, peaking at day-1 (TNF-α: 272.74 ± 60.31 pg/mL; IL-17: 37.00 ± 8.19 pg/mL; CRP: 194.57 ± 51.82 ng/mL; n = 11) and declining to their lowest values at day-30 (TNF-α: 179.82 ± 34.79 pg/mL; IL-17: 24.68 ± 4.50 pg/mL; CRP: 123.84 ± 26.94 ng/mL; n = 12). One-way ANOVA confirmed significant intergroup differences for TNF-α (F = 8.75, P < 0.001), IL-17 (F = 6.45, P = 0.002), and CRP (F = 8.63, P = 0.008; Table 2; Figure 5). Pairwise comparisons indicated statistically significant reductions from day-1 to day-30 for all three markers (P < 0.001 for each; Table 3). No significant differences were observed between day-30 and later time points (P > 0.05). Temporal trends of normalized inflammatory markers are illustrated in Figures 6, 7.

**TABLE 2:** Descriptive statistics for inflammatory and oxidative biomarkers by group.

Group	n	TNF-α (pg/mL)	IL-17 (pg/mL)	CRP (ng/mL)	MDA (nmol/mL)	SOD (ng/mL)
Day-1	11	272.74 ± 60.31	37.00 ± 8.19	194.57 ± 51.82	7.79 ± 1.36	92.18 ± 12.26
Day-10	11	214.12 ± 37.93	30.91 ± 4.66	125.71 ± 19.06	5.56 ± 1.71	96.06 ± 12.87
Day-20	12	190.21 ± 33.40	29.97 ± 4.69	120.27 ± 18.68	6.09 ± 1.08	95.54 ± 11.60
Day-30	12	179.82 ± 34.79	24.68 ± 4.50	123.84 ± 26.94	4.27 ± 0.97	91.10 ± 14.11
Day-40	12	175.55 ± 34.79	26.48 ± 5.52	127.56 ± 28.64	4.64 ± 0.35	95.32 ± 11.88
Day-50	12	175.35 ± 38.44	27.56 ± 5.43	128.64 ± 27.34	4.23 ± 0.25	93.23 ± 14.56
Day-60	12	180.55 ± 34.79	26.38 ± 4.52	130.56 ± 23.34	4.37 ± 0.75	96.92 ± 10.98

**TABLE 3:** Pairwise statistical comparisons for biomarkers (p-values).

Timepoints	TNF-α	IL-17	CRP	MDA	SOD
Day-1 vs. Day-10	0.002	0.016	<0.001	<0.001	0.375
Day-1 vs. Day-20	<0.001	0.005	<0.001	0.003	0.058
Day-1 vs. Day-30	<0.001	<0.001	<0.001	<0.001	0.987
Day-1 vs. Day-40	<0.001	<0.001	<0.001	<0.001	0.192
Day-1 vs. Day-50	<0.001	<0.001	<0.001	<0.001	0.157
Day-1 vs. Day-60	<0.001	<0.001	<0.001	<0.001	0.213
Day-10 vs. Day-20	0.023	0.036	0.044	0.049	0.309
Day-10 vs. Day-30	<0.001	<0.001	<0.001	<0.001	0.357
Day-10 vs. Day-40	<0.001	<0.001	<0.001	<0.001	0.678
Day-10 vs. Day-50	<0.001	<0.001	<0.001	<0.001	0.712
Day-10 vs. Day-60	<0.001	<0.001	<0.001	<0.001	0.593
Day-20 vs. Day-30	0.032	0.038	0.028	0.033	0.051
Day-20 vs. Day-40	0.035	0.042	0.039	0.029	0.823
Day-20 vs. Day-50	0.042	0.044	0.032	0.048	0.789
Day-20 vs. Day-60	0.046	0.642	0.047	0.907	0.654
Day-30 vs. Day-40	0.754	0.798	0.258	0.895	0.951
Day-30 vs. Day-50	0.652	0.712	0.268	0.951	0.962
Day-30 vs. Day-60	0.799	0.965	0.298	0.523	0.997
Day-40 vs. Day-50	0.998	0.952	0.997	0.912	0.976
Day-40 vs. Day-60	0.554	0.431	0.784	0.902	0.951
Day-50 vs. Day-60	0.999	0.971	0.999	0.614	0.995

### Oxidative stress marker profiles

**FIGURE 8:**
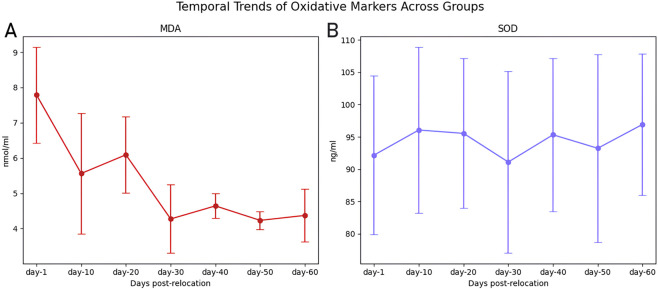
Temporal Trends of Oxidative Markers (MDA and SOD) Across Groups. Temporal trends of oxidative stress biomarkers in serum at different time points following relocation from high-altitude to normoxia. **(A)** Malondialdehyde (MDA) levels, an indicator of lipid peroxidation, significantly decreased over time, reaching a nadir at day-30 post-relocation. **(B)** Superoxide dismutase (SOD) activity, representing antioxidant capacity, remained relatively stable across groups. Data are presented as mean ± SD for each group (day-1, day-10, day-20, day-30, day-40, day-50, and day-60 post-relocation).

**FIGURE 9:**
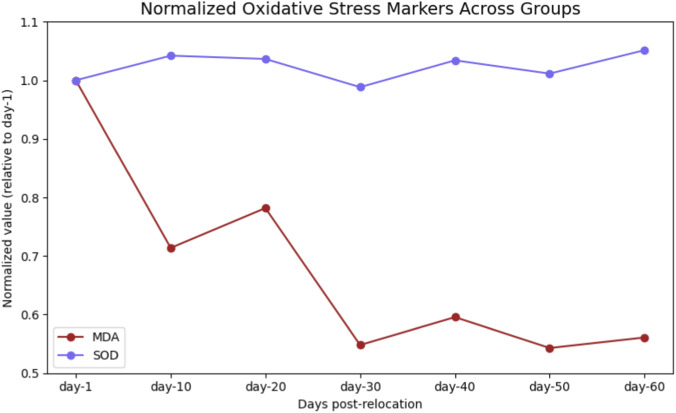
Overlay of Normalized Oxidative Stress Markers (MDA and SOD) Across Groups. Overlay of serum malondialdehyde (MDA) and superoxide dismutase (SOD) levels, each normalized to their respective day-1 value, across all experimental groups. MDA levels decreased substantially over time, reaching their lowest values at day-30 post-relocation, while SOD activity remained relatively stable. Data represent the relative change in oxidative stress marker concentration following relocation from high-altitude to normoxia and surgical intervention at each time point (day-1, day-10, day-20, day-30, day-40, day-50, and day-60).

MDA levels, indicative of lipid peroxidation, were highest at day-1 (7.79 ± 1.36 nmol/mL; n = 11) and decreased progressively to a nadir at day-30 (4.27 ± 0.97 nmol/mL; n = 12; P < 0.001; Table 2; Figure 8). Pairwise comparisons demonstrated significant reductions in MDA at each successive interval up to day-30 (all P < 0.05; Table 3). SOD activity remained stable across all groups (range: 91.10 ± 14.11 to 96.92 ± 10.98 ng/mL; P = 0.125), with no significant temporal differences detected (Table 2; Figures 8, 9).

### Immune cell infiltration and tissue repair indices

**FIGURE 10:**
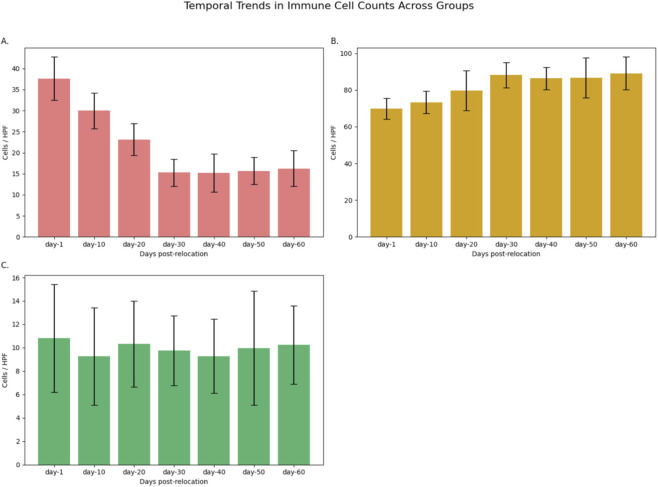
Temporal Trends in Immune Cell Counts Across Groups. Mean (±SD) counts of **(A)** macrophages, **(B)** fibroblasts, and **(C)** neutrophils per high-power field (HPF) in small bowel tissue at each time point post-relocation. Data are shown as bar plots with error bars representing standard deviation for each group (day-1, day-10, day-20, day-30, day-40, day-50, and day-60). Macrophage counts decreased significantly over time, while fibroblast numbers increased, peaking at day-30. Neutrophil counts remained relatively stable across groups.

**FIGURE 11:**
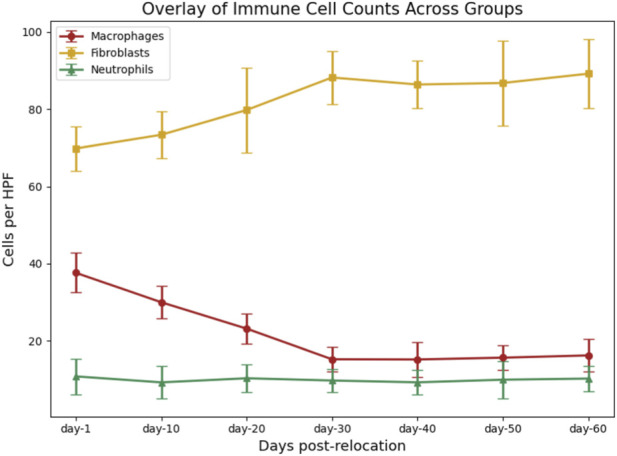
Overlay of Immune Cell Counts Across Groups. Overlay plot showing mean (±SD) counts of macrophages, fibroblasts, and neutrophils per high-power field (HPF) in small bowel tissue at each time point post-relocation. Macrophage numbers decreased significantly over time, fibroblast numbers increased with a peak at day-30, and neutrophil counts remained stable across groups. Error bars represent standard deviation for each group.

Quantification of cellular repair indices demonstrated significant differences among groups. Macrophage counts (CD68^+^ cells/HPF) were highest at day-1 (37.64 ± 5.14; n = 11), declining steadily to a minimum at day-30 (15.25 ± 3.25; n = 12; F = 52.38, P < 0.001; Table 4; Figure 10). Pairwise analyses confirmed that reductions from day-1 to day-30 were statistically significant (P < 0.001; Table 5). In contrast, fibroblast density (vimentin + cells/HPF) increased progressively, peaking at day-30 (88.17 ± 6.85; n = 12), with significant differences between early and later time points (F = 7.84, P < 0.001; Table 5; Figure 10). Neutrophil counts did not vary significantly across groups (P = 0.867; Table 5). These trends are further visualized in Figure 11.

**TABLE 4:** Descriptive statistics for immune cell counts by group.

Group	n	Macrophages (n/HPF)	Fibroblasts (n/HPF)	Neutrophils (n/HPF)
Day-1	11	37.64 ± 5.14	69.82 ± 5.74	10.82 ± 4.62
Day-10	11	30.00 ± 4.24	73.36 ± 6.09	9.27 ± 4.17
Day-20	12	23.17 ± 3.83	79.75 ± 10.97	10.33 ± 3.68
Day-30	12	15.25 ± 3.25	88.17 ± 6.85	9.75 ± 2.99
Day-40	12	15.20 ± 4.54	86.36 ± 6.09	9.28 ± 3.17
Day-50	12	15.67 ± 3.23	86.75 ± 10.97	9.96 ± 4.88
Day-60	12	16.25 ± 4.25	89.16 ± 8.92	10.25 ± 3.36

**TABLE 5:** Pairwise statistical comparisons for immune cell counts (p-values).

Time points	Macrophages	Fibroblasts
Day-1 vs. Day-10	<0.001	<0.001
Day-1 vs. Day-20	<0.001	<0.001
Day-1 vs. Day-30	<0.001	<0.001
Day-1 vs. Day-40	<0.001	<0.001
Day-1 vs. Day-50	<0.001	<0.001
Day-1 vs. Day-60	<0.001	<0.001
Day-10 vs. Day-20	<0.001	0.056
Day-10 vs. Day-30	<0.001	0.148
Day-10 vs. Day-40	<0.001	0.149
Day-10 vs. Day-50	<0.001	0.156
Day-10 vs. Day-60	<0.001	0.206
Day-20 vs. Day-30	<0.001	0.621
Day-20 vs. Day-40	<0.001	0.546
Day-20 vs. Day-50	<0.001	0.548
Day-20 vs. Day-60	<0.001	0.568
Day-30 vs. Day-40	0.908	0.424
Day-30 vs. Day-50	0.892	0.966
Day-30 vs. Day-60	0.936	0.732
Day-40 vs. Day-50	0.938	0.424
Day-40 vs. Day-60	0.822	0.926
Day-50 vs. Day-60	0.896	0.839

### Histopathological repair quality

**FIGURE 12:**
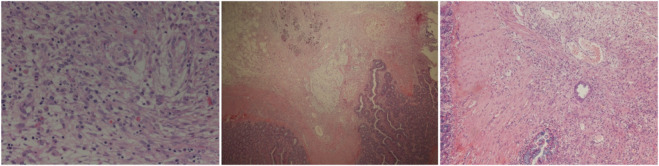
Temporal stratification of histopathological repair quality following high-altitude de-adaptation (in day-1、day-30 and day-60 groups).

Histological examination of small bowel repair sites revealed time-dependent improvements in tissue architecture. Day-1 specimens showed extensive inflammatory infiltration, immature granulation tissue, and marked edema. By day-30, there was a marked reduction in inflammation and edema, with well-organized granulation tissue and mature collagen deposition. Day-60 samples demonstrated mature granulation tissue and minimal residual inflammation, indicating a stabilized healing process (Figure 12).

### Integrated summary of key findings

**FIGURE 13:**
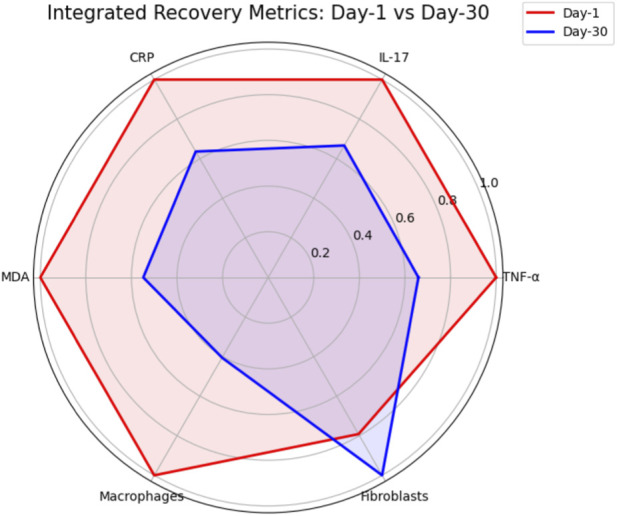
Integrated Recovery Metrics: Day-1 vs. Day-30. Radar plot comparing integrated recovery metrics between day-1 and day-30 post-relocation groups. Parameters include TNF-α, IL-17, and CRP (inflammatory markers), MDA (oxidative stress marker), and counts of macrophages and fibroblasts (cellular repair indices), normalized to the maximum value observed for each parameter. The plot illustrates a substantial reduction in inflammation, oxidative stress, and macrophage infiltration at day-30, with a concomitant increase in fibroblast numbers, indicating optimal tissue repair and recovery compared to day-1.

A comprehensive summary of outcomes at each time point is provided in Table 6. By day-30 post-relocation, animals demonstrated the lowest levels of systemic inflammation and oxidative stress, the lowest macrophage infiltration, and the highest fibroblast density, corresponding to optimal histopathological repair. This trend is further depicted in the integrated radar plot comparing day-1 and day-30 recovery metrics (Figure 13).

**TABLE 6:** Summary table of key findings by time point.

Timepoint	Inflammation	Oxidative stress	Macrophages	Fibroblasts	Survival	Overall healing (qualitative)
Day-1	Highest	Highest	Highest	Lowest	91.7%	Delayed/poor
Day-10	High	High	High	Low	91.7%	Suboptimal
Day-20	Moderate	Moderate	Moderate	Moderate	100%	Improving
Day-30	Lowest	Lowest	Lowest	Highest	100%	Optimal/robust
Day-40+	Low	Low	Low	High	100%	Maintained

## Discussion

This study systematically evaluated the temporal impact of high-altitude de-adaptation on small bowel repair outcomes in rats and identified a critical 30-day post-relocation window during which surgical intervention was associated with optimal tissue repair. Importantly, the present results extend prior observations by demonstrating that this window coincides with the coordinated resolution of hypoxia-induced physiological adaptations, systemic inflammatory activity, and oxidative stress, rather than reflecting an isolated local healing phenomenon.

A key prerequisite for interpreting de-adaptation–associated surgical vulnerability is confirmation that animals entered the postoperative phase from a well-defined hypoxia-adapted state. In this study, chronic exposure to simulated high altitude induced robust systemic and molecular adaptations, including sustained reductions in arterial oxygen saturation, enhanced erythropoiesis, increased respiratory rate, transient weight loss, and upregulation of intestinal HIF-1α and VEGF expression. The subsequent time-dependent normalization of these parameters after relocation to normoxia, with hematological indices and hypoxia-responsive signaling returning to baseline levels by approximately 30 days, provides direct physiological evidence that de adaptation is a gradual and quantifiable process rather than an immediate reversal of hypoxic exposure. These findings are consistent with clinical observations of elevated postoperative morbidity in high-altitude migrants undergoing surgery shortly after relocation and provide important pathophysiological context for understanding the interaction between de-adaptation–related physiological instability and surgical stress (Mrakic-Sposta et al., 2022; McGettrick and O'Neill, 2020).

Within this context, the biphasic patterns of inflammatory cytokines (TNF-α, IL-17, and CRP) and oxidative stress (MDA) strongly support a “dual-hit” model in which unresolved de-adaptation–related perturbations amplify the inflammatory burden imposed by surgical trauma. The marked elevation of cytokines and lipid peroxidation at day 1 post-relocation is consistent with acute hypoxia–reoxygenation stress and excessive mitochondrial reactive oxygen species generation, a phenomenon well documented in hypoxia and ischemia–reperfusion models (Apostolova and Victor, 2015; Rotariu et al., 2022). The progressive attenuation of these markers by day 30, in parallel with normalization of HIF-1α and VEGF expression and stable SOD activity, suggests restoration of redox homeostasis and immune equilibrium during normoxic re-acclimatization (Jaśkiewicz et al., 2022; Pham et al., 2021). This temporal convergence reinforces the biological plausibility of the 30-day window as a threshold for physiological stabilization.

From a broader physiological perspective, the de-adaptation process observed in this study shares notable similarities with other systemic stress syndromes, such as heat stroke, in which abrupt environmental change triggers a coordinated inflammatory and oxidative response (Baindara et al., 2025). Heat stroke is characterized by a systemic inflammatory response syndrome (SIRS), excessive cytokine release, oxidative injury, and secondary organ dysfunction, with the gastrointestinal tract being particularly vulnerable due to barrier disruption and immune activation (Baindara et al., 2025; Iba et al., 2025; Sun et al., 2024).

In this context, the early post-relocation phase of high-altitude de-adaptation may represent a comparable state of systemic physiological instability, in which reoxygenation acts as a triggering stressor rather than a benign normalization process. The convergence of heightened inflammatory activity, lipid peroxidation, and impaired tissue repair observed in the early timepoints of this study parallels key pathophysiological features described in heat stroke–associated intestinal injury models, thereby situating de-adaptation within a broader framework of stress-induced inflammatory dysregulation.

Consistent with this interpretation, histopathological and cellular analyses further support this interpretation by revealing a time-dependent shift in repair dynamics. Early postoperative timepoints were dominated by macrophage-rich inflammatory infiltrates, whereas the day-30 cohort demonstrated a marked reduction in macrophage density accompanied by peak fibroblast accumulation and more organized granulation tissue and collagen deposition. This transition from inflammation-dominated injury responses to stromal remodeling reflects a critical reparative inflection point during de-adaptation. Deviations from this temporal balance, occurring either too early when inflammation remains excessive or later when reparative activity plateaus, were associated with less optimal tissue architecture. These findings align with established roles of macrophages and fibroblasts in intestinal wound healing and underscore the importance of synchronizing surgical timing with the underlying repair milieu (Chalkidi et al., 2022; Yadav et al., 2024).

The absence of significant differences in perioperative mortality among groups indicates that short-term survival is relatively insensitive to the duration of de-adaptation. However, the improved functional recovery observed in the day-30 cohort, including normalized feeding behavior and activity levels, highlights the clinical relevance of surgical timing with respect to physiological stabilization following descent.

By integrating de-adaptation timelines with surgical outcomes, our study extends prior work on hypoxia–reoxygenation injury models (Wang et al., 2024; Huang et al., 2024). The stabilization of MDA levels after day 30 parallels observations from ischemia–reperfusion studies, in which attenuation of oxidative stress coincides with restoration of endothelial barrier function (He et al., 2020). Likewise, the biphasic cytokine patterns observed here resemble clinical reports of prolonged inflammatory activation in high-altitude migrants undergoing surgery soon after relocation (Shang et al., 2022; Khan et al., 2021). Together, these parallels suggest that approximately four to 6 weeks may represent a critical period for resolution of systemic hypoxia-related adaptations after descent, although organ-specific recovery trajectories remain incompletely defined.

The observed dissociation between MDA and SOD levels indicates that oxidative stress during de-adaptation may predominantly reflect increased reactive oxygen species generation rather than impaired antioxidant capacity (Liu et al., 2023; Tafani et al., 2016). While this observation raises the possibility that targeted modulation of oxidative stress during early de-adaptation could be beneficial, such interpretations remain speculative in the absence of direct functional intervention.

From a clinical perspective, current perioperative guidelines provide limited guidance for managing patients relocating from high-altitude environments. Conceptually, this gap mirrors challenges encountered in other systemic stress conditions, such as heat stroke or severe inflammatory syndromes, where the timing of surgical or invasive interventions relative to physiological stabilization critically influences outcomes. Our findings suggest that postponing elective abdominal surgery for approximately 30 days after descent may be associated with improved tissue repair outcomes, consistent with retrospective analyses linking delayed intervention to reduced postoperative morbidity in other settings (Tran et al., 2021). However, whether pharmacological modulation of inflammation or oxidative stress could mitigate risk in unavoidable early or emergency surgeries requires direct experimental validation.

Several limitations of the present study should be acknowledged. First, the exclusive use of male rats limits the generalizability of the findings, particularly in light of well documented sex related differences in hypoxia tolerance, inflammatory responses, and tissue repair mechanisms (Kelly et al., 2023; Alcantara-Zapata et al., 2022). Future studies incorporating female animals and sex stratified analyses will be necessary to determine whether the observed de adaptation dependent effects exhibit sex specific patterns. Second, the postoperative observation period was limited to 10 days, which precluded assessment of longer term outcomes such as stricture formation or postoperative adhesion development (Almog and Zani, 2021). In addition, although the hypobaric chamber model provides a controlled and reproducible simulation of high-altitude exposure, it cannot fully capture the environmental and physiological complexity of natural high altitude settings. Finally, the present study is observational in design and does not include targeted functional interventions aimed at modulating oxidative stress or inflammatory pathways. As a result, causal relationships between these biological processes and postoperative repair outcomes cannot be conclusively established. While comparisons with heat stroke and other systemic stress models offer valuable physiological context, the molecular drivers underlying de adaptation related injury may differ across conditions and will require condition specific validation in future investigations.

In summary, this preclinical study demonstrates that the timing of surgical intervention relative to the de-adaptation period is closely associated with intestinal repair outcomes following descent from high altitude. The identification of a 30-day post-relocation window characterized by reduced inflammatory and oxidative activity and improved histological repair provides a physiologically grounded framework for surgical decision-making in high-altitude migrants. Future studies incorporating targeted interventions and mechanistic analyses will be essential to establish causality and refine perioperative management strategies for this population.

## Data Availability

The original contributions presented in the study are included in the article/supplementary material, further inquiries can be directed to the corresponding authors.
